# Effectiveness of WeChat for Improving Exclusive Breastfeeding in Huzhu County China: Randomized Controlled Trial

**DOI:** 10.2196/23273

**Published:** 2020-12-03

**Authors:** Qiong Wu, Yiwen Huang, Zijun Liao, Michelle Helena van Velthoven, Wei Wang, Yanfeng Zhang

**Affiliations:** 1 Capital Institute of Pediatrics Beijing China; 2 University of Oxford Oxford United Kingdom

**Keywords:** breastfeeding, exclusive breastfeeding, WeChat, mHealth, randomized controlled trial

## Abstract

**Background:**

The benefits of breastfeeding for both infants and mothers have been well recognized. However, the exclusive breastfeeding rate in China is low and decreasing. Mobile technologies have rapidly developed; communication apps such as WeChat (one of the largest social networking platforms in China) are widely used and have the potential to conveniently improve health behaviors.

**Objective:**

This study aimed to assess the effectiveness of using WeChat to improve breastfeeding practices.

**Methods:**

This 2-arm randomized controlled trial was conducted among pregnant women from May 2019 to April 2020 in Huzhu County, Qinghai Province, China. Pregnant women were eligible to participate if they were aged 18 years or older, were 11 to 37 weeks pregnant with a singleton fetus, had no known illness that could limit breastfeeding after childbirth, used WeChat through their smartphone, and had access to the internet. A total of 344 pregnant women were recruited at baseline, with 170 in the intervention group and 174 in the control group. Women in the intervention group received breastfeeding knowledge and promotion information weekly through a WeChat official account from their third month of pregnancy to 6 months postpartum. The primary outcome of exclusive and predominant breastfeeding rate was measured 0-1 month, 2-3 months, and 4-5 months postpartum.

**Results:**

At 0-1 month postpartum, the exclusive breastfeeding rate was significantly higher in the intervention group than that in the control group (81.1% vs 63.3%; odds ratio [OR] 2.75, 95% CI 1.58-4.78; *P*<.001). Similarly, mothers in the intervention group were more likely to provide predominantly breast milk (OR 2.77, 95% CI 1.55-4.96; *P*<.001) and less likely to give dairy products to their children (OR 0.40, 95% CI 0.21-0.75; *P*=.005). There was no statistically significant difference for exclusive breastfeeding rate 2-3 months (*P*=.09) and 4-5 months postpartum (*P*=.27), though more children in the intervention group were exclusively breastfed than those in the control group 2-3 months postpartum (intervention: 111/152, 73.0%; control: 96/152, 63.2%) and 4-5 months postpartum(intervention: 50/108, 46.3%; control: 46/109, 42.2%).

**Conclusions:**

This study is the first effort to promote exclusive breastfeeding through WeChat in China, which proved to be an effective method of promoting exclusive breastfeeding in early life. WeChat health education can be used in addition to local breastfeeding promotion programs.

**Trial Registration:**

Chinese Clinical Trial Registry ChiCTR1800017364; http://www.chictr.org.cn/showproj.aspx?proj=29325

**International Registered Report Identifier (IRRID):**

RR2-10.1186/s12889-019-7676-2

## Introduction

Appropriate child feeding is the foundation for good nutritional intake and healthy development and is a critical factor for health in adults [1-3]. As a part of optimal feeding practices, exclusive breastfeeding is recognized as a cornerstone of child survival and health, by providing essential irreplaceable nutrition for a child’s growth and development [4]. Therefore, the World Health Organization (WHO) and United Nations Children’s Fund (UNICEF) recommend that children should be exclusively breastfed from birth to 6 months of age and continually breastfed until they are 2 years old or older [5]. Moreover, one of the WHO global nutrition targets for 2025 is increasing the exclusive breastfeeding rate in the first 6 months of life to at least 50% [4].

In China, the exclusive breastfeeding rate for under 6 months of age is a concern and achieving the 2025 global target remains an ongoing challenge. According to the Chinese national data, the exclusive breastfeeding rate under 6 months was 27.6% in 2008 [6] and 20.7% in 2013 (the weighted exclusive breastfeeding rate was 18.6%) [7], which showed a downward trend. Therefore, much effort is required to explore effective ways to control this downward trend, and hence, to promote breastfeeding in China.

Positive breastfeeding outcomes are usually related to the improvement of maternal breastfeeding knowledge and attitudes [2,8]. Although interventions on breastfeeding promotion vary worldwide, education and support are the 2 most common approaches, and interventions on exclusive breastfeeding containing these 2 elements have mixed success [9,10]. In China, breastfeeding education is generally implemented through the rural 3-tier health care system (county-township-village) at antenatal and postnatal care [11]. Since 2009, China has been implementing a national program called Basic Public Health Service, in which health care workers are required to provide face-to-face breastfeeding and complementary feeding counseling to pregnant women and mothers throughout antenatal and postnatal care [11]. However, research has indicated that mothers in rural areas rarely receive feeding information from health facilities; their main sources of information were family members and friends, who were unlikely to have access to better information and may have misinformed mothers [12-14]. Therefore, new channels are needed in rural China to deliver effective infant feeding education.

With the widespread use of smartphones, using apps in the health sector for delivering health care services and health promotion is an increasing phenomenon [15-19]. In China, one of the most popular smartphone apps is WeChat, which offers services such as Facebook, Twitter, WhatsApp, and others, on a single platform. More than 1.32 billion users were registered with WeChat throughout the world by Q2 2019, and more than 1.15 billion people were monthly active users [20]. Approximately 45 billion messages are exchanged on the platform every day [20]. Furthermore, there is an app-within-an-app platform in WeChat called WeChat Official Accounts, which can be used for individuals, governments, media organizations, and business enterprises to communicate and interact with their subscribers and provide them with services through text, images, voice, videos, and rich-media messages [21]. There were more than 20 million registered WeChat official accounts at the end of 2018 [20]. WeChat is gradually changing the channels through which people receive information and has been used as a communication tool to change health behaviors, showing potential positive impacts on disease management of cancer [22], malaria [23], asthma [24], chronic rhinosinusitis [25], diabetes [26], and weight loss [27]. However, no studies have focused on using WeChat to support caregivers with infant and young child feeding. The objective of this randomized controlled trial was to evaluate the effectiveness of a WeChat breastfeeding intervention on promoting exclusive breastfeeding in rural areas of China.

## Methods

### Study Design

A 2-arm randomized controlled trial (Chinese Clinical Trial Registry ChiCTR1800017364) was conducted between May 9, 2019 and April 3, 2020. We aimed to evaluate the effectiveness of using a WeChat account for improving exclusive breastfeeding of children aged 0-5 months. The protocol for this randomized controlled trial was previously published [28]. The sampling unit was individual pregnant women, who were randomized to routine antenatal and postnatal care or routine care plus the WeChat breastfeeding education.

### Study Sites and Context

This trial was carried out in 13 townships in Huzhu County, Qinghai Province, China. Qinghai Province is in northwest China, with a total population of 6,078,200 in 2019. Huzhu County lies in the northeast of Qinghai province and has a total population of 401,540 [29]. There were 91,321 women of reproductive age and 4325 pregnant women in Huzhu County in 2017 (Huzhu County Maternal and Child Health Family Planning Service Centre).

Huzhu County has 19 townships and 294 villages. We excluded 6 townships; 4 townships had already been selected by another maternal and child health project, and the other 2 were remote with a small number of pregnant women.

### Participants and Recruitment

Pregnant women were eligible to participate in this trial if they were aged at least 18 years, were 11-37 weeks pregnant with a singleton fetus, had no known illness that limits breastfeeding after childbirth, were able to read and communicate in Mandarin, used WeChat through their smartphone, and had access to the internet. The exclusion criteria were (1) pregnant women who did not come to the township hospitals to participate in the trial; (2) pregnant women with severe disease and complications of pregnancy or HIV; (3) women who had a miscarriage or stillbirth; (4) mothers with infants with a low birth weight (<2500 g) or who were born prematurely (<37 weeks of gestation).

Before recruitment, we asked each township hospital to provide a list of the names of all pregnant women between 11 to 37 weeks’ gestation, which included information on gestational age, gravidity, and parity. A total of 444 women were listed. Based on this list, participating pregnant women were invited to come to their township hospitals and were given full information about the study between May 9, 2019 and May 17, 2019. After agreeing to participate and signing the written consent form, a researcher gave each eligible pregnant woman an opaque sealed envelope, which included a random number generated in advance and indicated the allocated group. After completing baseline data collection, all the eligible participants were randomized to either the WeChat intervention group or the control group at a 1:1 ratio.

### Sample Size Calculation

The sample size for this study was estimated using a 0-5 months of age exclusive breastfeeding rate of 29.2% from a pilot cross-sectional survey conducted by our team in Datong County in Qinghai in September 2017. We expected to achieve a 20% increase in the exclusive breastfeeding rate with the WeChat intervention. Assuming a power of 80% and a 5% significance level, we determined that a sample size of 93 pregnant women for each (intervention and control) group was needed for this study. To compensate for attrition and loss to follow-up, we planned to enroll 200 pregnant women in each group.

### Intervention Group

Women in both intervention and control groups were asked to follow the WeChat account called Huzhu County Maternal and Child Health Family Planning Service Centre on their smartphone by scanning the 2D code at the back of random number cards. There was a special module called *Ke Xue Wei Yan*g (*Optimal Feeding*) within the WeChat Official Account which was developed by an information technology company, ZYZY (Beijing) Pioneer of Cultural Essence Co Ltd and pretested in Huzhu County in Aug 2018 [28]. Pregnant women allocated to the intervention group were also asked to subscribe to and register with the *Ke Xue Wei Yang* module by entering information on their name, phone number, gestational age, expected date of delivery, village and county of residence and were provided a WeChat log-in and password. Pregnant women in the control group were not able to register with the *Ke Xue Wei Yang* module and did not have access to the information in the module, to preclude contamination between groups through direct sharing of messages sent via WeChat.

**Figure 1:**
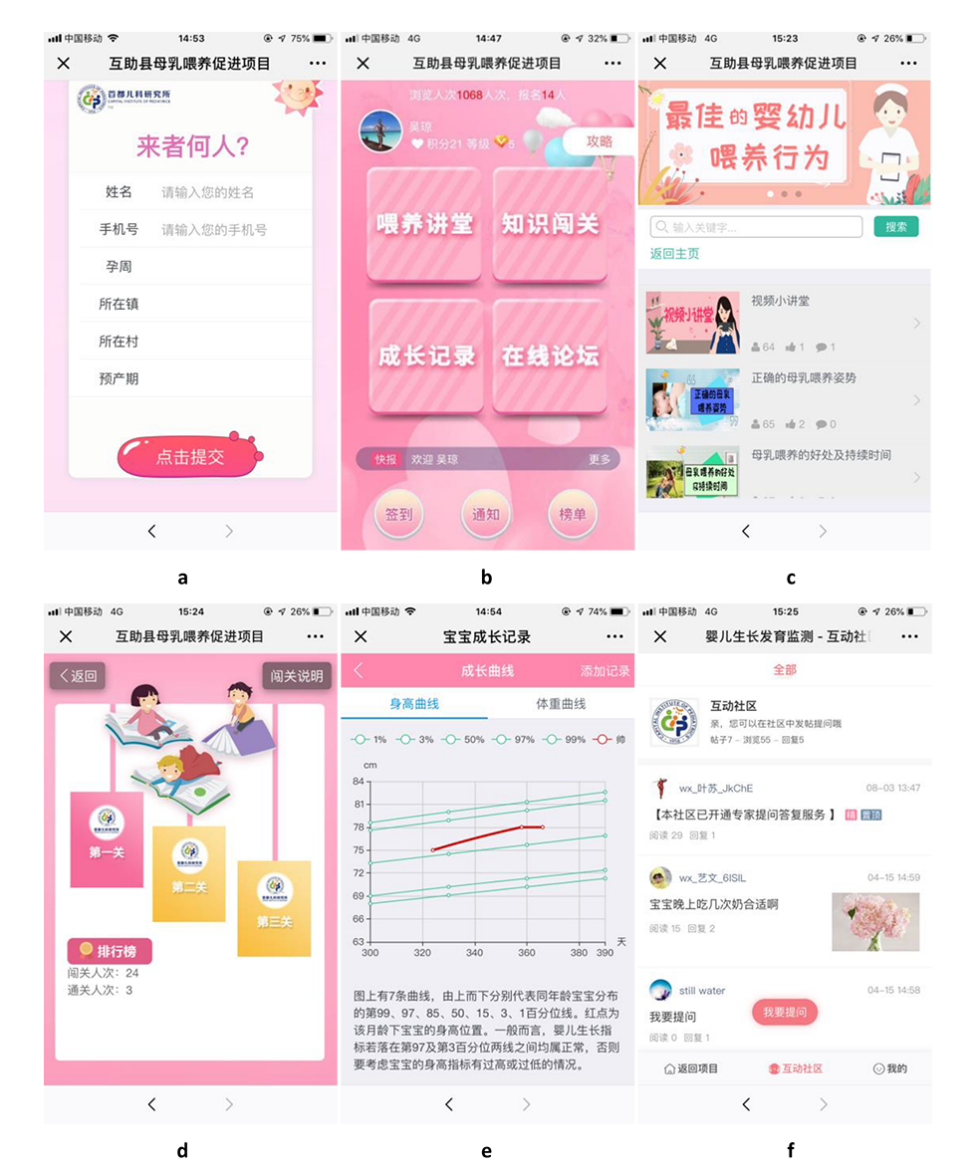
The interface of the WeChat intervention: (a) log-in interface, (b) main interface, (c) feeding messages, (d) feeding competition, (e) baby growth chart, and (f) online forum.

There were 4 components in the *Ke Xue Wei Yang* module: feeding messages, a feeding knowledge competition, a baby growth chart, and an online forum (Figure 1), which were described in detail in our protocol [28].

The feeding messages, the most important component, were designed for breastfeeding promotion education and provided key breastfeeding knowledge and relevant infant feeding advice, breastfeeding problems encountered for both mother and child, and preparation for both breastfeeding and complementary feeding. All messages were developed based on the WHO breastfeeding recommendations, guidelines, or published literature; messages were published before recruitment. Once women in the intervention group subscribed to the *Ke Xue Wei Yang* module, they could read all messages whenever they wanted. Furthermore, we considered that late pregnancy (37 weeks or above), the first month postpartum, and 4 months postpartum were 3 key stages for mothers and that breastfeeding information needed to be strengthened during these stages, so we sent an additional 3 sets of tailored messages to each pregnant woman and mother at these stages via WeChat on Monday, Wednesday, and Friday every week. Specifically, we sent information on getting ready for breastfeeding and key breastfeeding recommendations to women who were at least 37 weeks pregnant. For new mothers 1 month postpartum, we sent key breastfeeding recommendations and common breastfeeding problems encountered for both mother and child. We sent information on starting complementary feeding by 6 months of age to mothers whose children were 4 months or older (to avoid having mothers introduce complementary food too early or too late).

Women could participate in the feeding knowledge competition component to test their breastfeeding knowledge. Moreover, women could enter their children's weight and height in the baby growth chart component whenever they want to monitor their children’s growth and ask breastfeeding related questions on the online forum component.

### Data Collection

Data were collected by face-to-face at baseline and by telephone during follow-up interviews. Baseline data collection was carried out after the recruitment session. After receiving consent to participate, we interviewed the pregnant women (baseline questionnaire), which included information on demographic characteristics, antenatal care, hemoglobin levels, as well as breastfeeding knowledge. Baseline interviewers, who were from a vocational-technical school in Huzhu County and trained on interview methodology, used smartphones to collect the baseline data.

Follow-up interviews were conducted by telephone at 0-1 months (mean 35.3 days, SD 10.5), 2-3 months (mean 102.7 days, SD 9.9), 4-5 months (mean 157.2 days, SD 17.8) postpartum. Data from women in both the intervention group and the control group were collected on breastfeeding knowledge, practices, reasons for weaning, and information channels by health workers form Huzhu County Maternal and Child Health Family Planning Service Centre, who were blinded to intervention status and trained on interview methodology by research staff from the Capital Institute of Pediatrics.

### Outcome Measurement

The primary outcome measure was exclusive and predominant breastfeeding rate at 0-1 month (0-60 days), 2-3 months (61-120 days), and 4-5 months (121-180 days) postpartum in both the intervention group and the control group. Secondary outcomes included the following: (1) the proportion of early initiation of breastfeeding; (2) prelacteal feeding rate; (3) rate of any breastfeeding; (4) mothers’ knowledge on breastfeeding practices; (5) other infant feeding practices (such as giving dairy or dairy products, water, semisolid, or solid foods at 3 follow-ups).

According to the WHO definition, *exclusive breastfeeding* is defined as an infant receiving only breast milk, no other liquids or solids, except oral rehydration solution, drops or syrups for vitamins, minerals supplementation, or medicine [30]; *predominant breastfeeding* permits partial substitution with water-based fluids; and *early initiation of breastfeeding* is when an infant is put to the mother’s breast within 1 hour of birth [30]. *Prelacteal feeding* was defined as the newborn being provided any food except mother’s milk before initiating breastfeeding; *any breastfeeding* included partial substitution with infant formula, other fluids, or solid foods.

### Data Management and Analysis

We performed statistical analysis with SAS (version 9.2 for Windows; SAS Institute). We summarized baseline characteristics, follow-up infant feeding practices, and mother’s infant feeding knowledge, as median and interquartile range (IQR) for continuous variables or as number and proportion for categorical variables. We estimated the homogeneity in baseline characteristics between groups using the Wilcoxon rank-sum test for nonparametric continuous variables, and chi-square or Fisher exact test for categorical variables. Multiple logistic regression models were used to estimate the effects of the intervention on breastfeeding rates between groups at the 3 follow-up points, controlling for parity at baseline, as the distribution of parity was different between the 2 groups. We also used chi-square tests to compare the differences in mother’s infant feeding knowledge between groups at baseline and 3 follow-up points. Participants who dropped out or who were lost to follow-up during the postpartum period were excluded from analysis and are reported separately (Multimedia Appendix 1). A *P* value <.05 was considered statistically significant.

### Ethics Statement

This study was approved by the ethical committee of the Capital Institute of Pediatrics, Beijing, China. All women were given an information sheet, and participating women provided both verbal and written informed consent.

## Results

### General

We recruited 344 pregnant women. We randomized 170 to the intervention group and 174 to the control group between May 9, 2019 and May 17, 2019; 25 were excluded due to miscarriage, stillbirth, premature birth, or low birth weight, leaving 319 participants for analysis (161 in the intervention group and 158 in the control group). A total of 32, 15, and 8 participants could not be contacted by phone for unknown reasons at the first, second, and third follow-ups, respectively. Moreover, 94 mothers missed the follow-up deadline (children <180 days age) at the 4-5 months postpartum follow-up because of the long holiday of Chinese Lunar New Year and the coronavirus (COVID-19) outbreak in January 2020 in China. The CONSORT (Consolidated Standards of Reporting Trials) [31] flow diagram is shown in Figure 2. Most demographic characteristics were similar between the participants who dropped out and participants who were followed up (Multimedia Appendix 1). However, at the 0-1 month postpartum follow-up, more mothers who only attended primary school dropped out; at the 4-5 months postpartum follow-up, more mothers who had a higher gestational age at enrollment dropped out.

**Figure:**
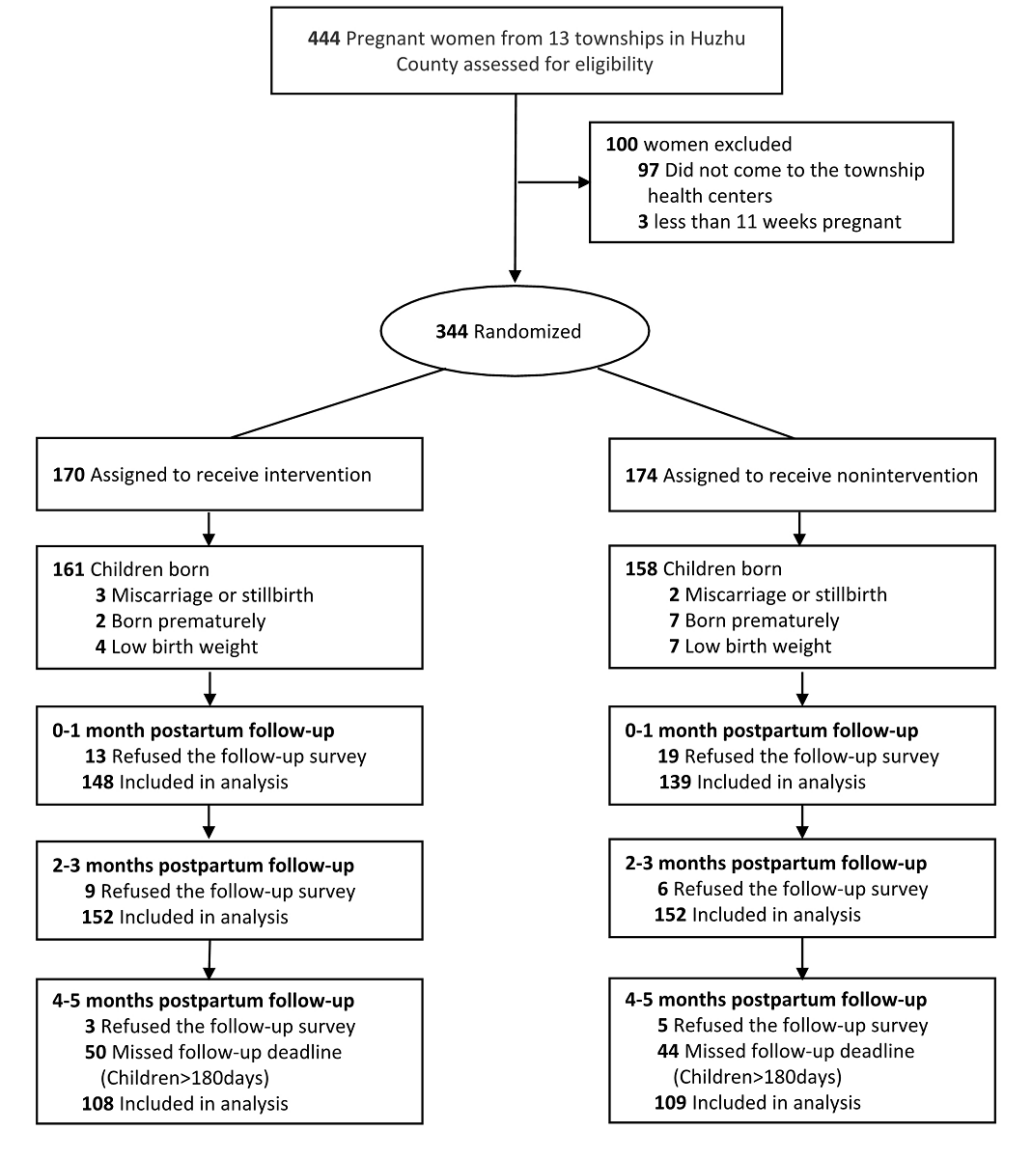
CONSORT flow diagram. CONSORT: Consolidated Standards of Reporting Trials.

### Characteristics of the Participants

The median age of enrolled participants was 28 years (IQR 25-31). There were no differences between the intervention and control groups for most demographic characteristics, except for gravidity and parity (Table 1). Compared with those in the control group, more participants in the intervention group had their first pregnancy (intervention: 46/161, 28.6%; control: 26/158, 16.5%; *P=*.01) or primipara (intervention: 48/161, 29.8%; control: 28/158, 17.7%; *P=*.01). The prevalence of maternal anemia was lower in the intervention group (64/161, 40.0%) than that in the control group (72/158, 45.6%), but the difference was not statistically significant (*P=*.32).

**Table 1:** Baseline characteristics of participants by treatment groups.

Characteristics		Total (N=319), n (%)	WeChat group (n=161), n (%)	Control group (n=158), n (%)	*P* value
Age (years), median (IQR)		28 (25-31)	28 (24-31)	28 (25-31)	.45
**Gestational age (weeks)**					**.27**
	11-27	186 (58.3)	89 (55.3)	97 (61.4)	
	28-42	133 (41.7)	72 (44.7)	61 (38.6)	
**Gravidity**					**.01**
	First pregnancy	72 (22.6)	46 (28.6)	26 (16.5)	
	Second pregnancy or more	247 (77.4)	115 (71.4)	132 (83.5)	
**Parity**					**.01**
	Primipara	76 (23.8)	48 (29.8)	28 (17.7)	
	Multipara	243 (76.2)	113 (70.2)	130 (82.3)	
**Education level**					**.62**
	Primary school or below	54 (16.9)	24 (14.9)	30 (19.0)	
	Middle school	173 (54.2)	89 (55.3)	84 (53.2)	
	High school or above	92 (28.9)	48 (29.8)	44 (27.8)	
**Occupation**					**.75**
	Stay-at-home	278 (87.1)	138 (85.7)	140 (88.6)	
	Self-employed	9 (2.8)	5 (3.1)	4 (2.5)	
	Farmer	5 (1.6)	2 (1.3)	3 (1.9)	
	Others	27 (8.5)	16 (9.9)	11 (7.0)	
**Nationality**					**.86**
	Han	244 (76.5)	125 (77.6)	119 (75.3)	
	Tu	49 (15.4)	23 (14.3)	26 (16.5)	
	Others	13 (8.1)	13 (8.1)	13 (8.2)	
Ever received antenatal care		252 (79.0)	121 (75.2)	131 (82.9)	.12
Anemia		136 (42.8)	64 (40.0)	72 (45.6)	.32

### WeChat Activity

A total of 108 messages were published in the *Ke Xue Wei Yang* module in the WeChat official account, which was read more than 8892 times. The top 5 read messages were (1) benefits of breastfeeding (393 times); (2) importance of the early initiation (284 times); (3) breastfeeding positions and latching-on (242 times); (4) what is early initiation (214 times); (5) WHO recommendation: children should be exclusively breastfed from birth to 6 months (214 times).

### Breastfeeding Practice and Knowledge

As illustrated in Table 2, nearly all children were breastfed in both groups. The early initiation rate was low (intervention: 93/148, 62.8%; control: 101/139, 72.7%); however, the difference was not statistically significant (*P=*.08). In addition, approximately 40% of children were given prelacteal feeding in both groups, with 25.8% (74/287) given infant formula and 16.7% (48/287) given water.

**Table 2:** Comparison of infant feeding practices.

Indicators			Total^a^		Intervention group^a^		Control group^a^		*P* value		Odds ratio (95% CI)		Adjusted odds ratio (95% CI)^b^	
**First follow-up (0-1 month)**			**287 (100)**		**148 (100)**		**139 (100)**							
	Early initiation		194 (67.6)		93 (62.8)		101 (72.7)		.11		0.64 (0.39-1.05)		0.66 (0.40-1.09)	
	Ever breastfeeding		283 (98.6)		147 (99.3)		136 (97.8)		.30		3.24 (0.33-31.54)		3.42 (0.34-34.4)	
	**Prelacteal feeding**		**122 (42.5)**		**59 (39.9)**		**63 (45.3)**		**.28**		**0.80 (0.50-1.28)**		**0.77 (0.48-1.24)**	
		Giving formula		74 (25.8)		37 (25.0)		37 (26.6)		N/A^c^		N/A		N/A
		Giving water		48 (16.7)		22 (14.9)		26 (18.7)		N/A		N/A		N/A
	Exclusive breastfeeding		208 (72.5)		120 (81.1)		88 (63.3)		<.001		2.49 (1.45-4.25)		2.75 (1.58-4.78)	
	Predominant breastfeeding		218 (76.0)		124 (83.8)		94 (67.6)		<.001		2.47 (1.41-4.34)		2.77 (1.55-4.96)	
	Any breastfeeding		279 (97.2)		146 (98.6)		133 (95.7)		.18		3.29 (0.65-16.59)		3.09 (0.61-15.79)	
	Giving water during the past 24 hours		17 (5.9)		6 (4.1)		11 (7.9)		.17		0.49 (0.18-1.34)		0.49 (0.17-1.35)	
	Giving dairy or dairy products during the past 24 hours		55 (19.2)		20 (13.5)		35 (25.2)		.005		0.46 (0.25-0.85)		0.40 (0.21-0.75)	
	Giving semisolid or solid foods during the past 24 hours		2 (0.7)		2 (1.4)		0		.17		N/A		N/A	
**Second follow-up (2-3 months)**			**304 (100)**		**152 (100)**		**152 (100)**							
	Exclusive breastfeeding		207 (68.1)		111 (73.0)		96 (63.2)		.09		1.47 (0.91, 2.38)		1.53 (0.94, 2.49)	
	Predominant breastfeeding		212 (69.7)		113 (74.3)		99 (65.1)		.07		1.55 (0.95, 2.54)		1.60 (0.96, 2.64)	
	Any breastfeeding		287 (94.4)		144 (94.7)		143 (94.1)		.64		1.13 (0.43-3.02)		1.27 (0.47-3.46)	
	Giving water during the past 24 hours		11 (3.6)		3 (2.0)		8 (5.3)		.18		0.36 (0.09-1.39)		0.40 (0.10-1.55)	
	Giving dairy or dairy products during the past 24 hours		66 (21.7)		28 (18.4)		38 (25.0)		.14		0.68 (0.39-1.18)		0.66 (0.38-1.15)	
	Giving semisolid or solid foods during the past 24 hours		5 (1.6)		2 (1.3)		3 (2.0)		.54		0.66 (0.10-4.02)		0.56 (0.09-3.56)	
**Third follow-up (4-5 months)**			**217 (100)**		**108 (100)**		**109 (100)**							
	Exclusive breastfeeding		96 (44.2)		50 (46.3)		46 (42.2)		.27		1.18 (0.69-2.02)		1.37 (0.78-2.39)	
	Predominant breastfeeding		111 (51.2)		58 (53.7)		53 (48.6)		.20		1.23 (0.72-2.09)		1.46 (0.82-2.51)	
	Any breastfeeding		198 (91.2)		101 (93.5)		97 (89.0)		.10		1.78 (0.68-4.72)		2.34 (0.84-6.54)	
	Giving water during the past 24 hours		27 (12.4)		12 (11.1)		15 (13.8)		.63		0.78 (0.34-1.76)		0.82 (0.36-1.87)	
	Giving dairy or dairy products during the past 24 hours		62 (28.6)		28 (25.9)		34 (31.2)		.12		0.77 (0.43-1.39)		0.61 (0.32-1.14)	
	Giving semisolid or solid foods during the past 24 hours		49 (22.6)		25 (23.2)		24 (22.0)		.94		1.07 (0.56-2.02)		1.02 (0.53-1.96)	

^a^The number of participants varied because of loss to follow-up.

^b^Multiple logistic regression controlled for baseline parity.

^c^N/A: not applicable.

There was a downward trend for exclusive breastfeeding, predominant feeding, and any breastfeeding across the 3 follow-ups. At the 0-1 month postpartum follow-up, the exclusive breastfeeding rate was significantly higher in the intervention group (120/148, 81.1%) than in the control group (88/139, 63.3%), with an odds ratio (OR) of 2.75 (95% CI 1.58-4.78; *P<*.001). Similarly, mothers in the intervention group were more likely to provide breast milk predominantly (intervention: 124/148, 83.8%; control: 94/139, 67.6%; OR 2.77, 95% CI 1.55-4.96; *P<*.001), and less likely to give dairy or dairy products to their children (intervention: 20/148, 13.5%; control: 35/139, 25.2%; OR 0.40, 95% CI 0.21-0.75; *P=*.005).

At the 2-3 months postpartum follow-up, exclusive breastfeeding (intervention: 111/152, 73.0%; control: 96/152, 63.2%) and predominant feeding (intervention: 113/152, 74.3%; control: 99/152, 65.1%) in the intervention group were higher than in the control group, and fewer children were given dairy or dairy products in the intervention group (intervention: 28/152, 18.4%; control: 38/152, 25.0%). However, the differences between the 2 groups were not statistically significant (*P=*.09 for exclusive breastfeeding, *P=*.07 for predominant feeding, and *P=*.14 for giving dairy or dairy products).

At 4-5 months postpartum, both the rates of exclusive breastfeeding and predominant feeding dropped to 44.2% (96/217) and 51.2% (111/217), respectively, and the proportion of children who were given dairy or dairy products increased to 28.6% (62/217) for both groups; there were no significant differences between the 2 groups (*P=*.27 for exclusive breastfeeding, *P=*.20 for predominant feeding, and *P=*.12 for giving dairy or dairy products).

At the first 2 follow-ups, the rate of any breastfeeding was quite high, whereas very few children were given water and semisolid or solid foods. However, any breastfeeding dropped to around 90% and the proportion of children who were given water and semisolid or solid foods increased to 12.4% (27/217) and 22.6% (49/217), respectively, 4-5 months postpartum. There were no significant differences between the 2 groups (0-1 month postpartum: *P*=.18 for any breastfeeding rate, *P*=.17 for giving water, and *P*=0.17 for giving semisolid or solid foods; 2-3 months postpartum: *P*=.64 for any breastfeeding rate, *P*=.18 for giving water, and *P*=.54 for giving semisolid or solid foods; 4-5 months postpartum: *P*=.10 for any breastfeeding rate, *P*=.63 for giving water, and *P*=.94 for giving semisolid or solid foods).

Table 3 shows that all feeding knowledge indicators were low at baseline, with 33.9% (108/319) knowing early initiation of breastfeeding, 28.8% (92/319) knowing the duration of exclusive breastfeeding, 1.3% (4/319) knowing continued breastfeeding until 2 years of age, and 61.8% (197/319) knowing introduction of complementary foods at 6-8 months in both groups. Mothers’ feeding knowledge was greatly improved at follow-ups (*P<*.001). However, there were no differences between the intervention group and the control group in baseline and each follow-up. The proportion of pregnant women who ever received breastfeeding information during pregnancy in both groups increased dramatically from only 25.7% (82/319) at baseline to more than 80%-90% of mothers who ever received breastfeeding information during pregnancy or after delivery (*P<*.001), however, there was also no difference between the 2 groups (0-1 month postpartum: *P*=.24, 2-3 months postpartum: *P*=.63, 4-5 months postpartum: *P*=.09). In addition, 16.3% (52/319) of pregnant women reported having ever received infant formula information during their pregnancy.

**Table 3:** Comparison of infant feeding knowledge between the intervention and control groups.

Outcomes		Groups			*P* value		
		All	Intervention	Control	Intervention vs control	Intervention, baseline vs follow-up	Control, baseline vs follow-up
**Baseline**		**319 (100)**	**161 (100)**	**158 (100)**			
	Knowing early initiation of breastfeeding	108 (33.9)	58 (36.0)	50 (31.6)	.41	N/A^a^	N/A
	Knowing the duration of exclusive breastfeeding	92 (28.8)	47 (29.2)	45 (28.5)	.89	N/A	N/A
	Knowing continued breastfeeding until 2 years	4 (1.3)	2 (1.2)	2 (1.3)	.98	N/A	N/A
	Knowing introduction of complementary foods at 6-8 months	197 (61.8)	101 (62.7)	96 (60.8)	.72	N/A	N/A
	Women ever received breastfeeding information during pregnancy	82 (25.7)	41 (25.5)	41 (25.9)	.92	N/A	N/A
	Women ever received infant formula information during pregnancy	52 (16.3)	29 (18.0)	23 (14.6)	.40	N/A	N/A
**First follow-up (0-1 month)**		**287 (100)**	**148 (100)**	**139 (100)**			
	Knowing early initiation of breastfeeding	196 (68.3)	101 (68.2)	95 (68.4)	.99	<.001	<.001
	Knowing the duration of exclusive breastfeeding	199 (69.3)	107 (72.3)	92 (66.2)	.26	<.001	<.001
	Knowing continued breastfeeding until 2 years	47 (16.4)	24 (16.2)	23 (16.6)	.94	<.001	<.001
	Knowing introduction of complementary foods at 6-8 months	240 (83.6)	123 (83.1)	117 (84.2)	.81	<.001	<.001
	Women ever received breastfeeding information during pregnancy or after delivery	239 (83.3)	127 (85.8)	112 (80.6)	.24	<.001	<.001
	Women ever received infant formula information during pregnancy or after delivery	14 (4.9)	9 (6.1)	5 (3.6)	.33	.001	.001
**Second follow-up (2-3 months)**		**304 (100)**	**152 (100)**	**152 (100)**			
	Knowing early initiation of breastfeeding	216 (71.1)	115 (75.7)	101 (66.5)	.08	<.001	<.001
	Knowing the duration of exclusive breastfeeding	240 (78.9)	122 (80.3)	118 (77.6)	.57	<.001	<.001
	Knowing continued breastfeeding until two years	86 (28.3)	44 (28.9)	42 (27.6)	.80	<.001	<.001
	Knowing introduction of complementary foods at 6-8 months	262 (86.2)	136 (89.5)	126 (82.9)	.10	<.001	<.001
	Women ever received breastfeeding information during pregnancy or after delivery	286 (94.1)	144 (94.7)	142 (93.4)	.63	<.001	<.001
	Women ever received infant formula information during pregnancy or after delivery	10 (3.3)	3 (2.0)	7 (4.6)	.20	<.001	.003
**Third follow-up (4-5 months)**		**217 (100)**	**108 (100)**	**109 (100)**			
	Knowing early initiation of breastfeeding	171 (78.8)	84 (77.8)	87 (79.8)	.71	<.001	<.001
	Knowing the duration of exclusive breastfeeding	194 (89.4)	94 (87.0)	100 (91.7)	.26	<.001	<.001
	Knowing continued breastfeeding until 2 years	80 (36.9)	44 (40.7)	36 (33.0)	.24	<.001	<.001
	Knowing introduction of complementary foods at 6-8 months	205 (94.5)	102 (94.4)	103 (94.5)	.99	<.001	<.001
	Women ever received breastfeeding information during pregnancy or after delivery	208 (95.9)	101 (93.5)	107 (98.2)	.09	<.001	<.001
	Women ever received infant formula information during pregnancy or after delivery	7 (3.2)	3 (2.8)	4 (3.7)	.71	<.001	.004

^a^N/A: not applicable.

## Discussion

### Principal Findings

This is the first randomized controlled trial using WeChat to promote exclusive breastfeeding in rural China. This study showed that antenatal plus postnatal WeChat breastfeeding education was associated with higher rates of exclusive and predominant breastfeeding in the early postnatal period. Mothers in the WeChat group had 2.7 times the odds of exclusive and predominant breastfeeding during the first 2 months postpartum. We found that giving dairy or dairy products, water, and semisolid or solid foods were common reasons for nonexclusive breastfeeding and that giving dairy or dairy products was the predominant reason, with 19.2% (55/287), 21.7% (66/304), and 28.6% (62/217) of all children being given dairy or dairy products (0-1 months, 2-3 months, and 4-5 months postpartum, respectively). In addition, 25.8% of newborns (74/287) were given prelacteal infant formula, which can limit an infant's frequency of suckling and expose them to increased risk of infection [32]. Our study demonstrated that a WeChat intervention could significantly reduce dairy or dairy product supplementation in early life, and thus improve the exclusive breastfeeding rate. The proportion in the WeChat group giving dairy or dairy products 0-1 month postpartum was significantly lower than those in the control group (*P*=.005). Although there was no significant difference between the groups 2-3 months (*P*=.14) or 4-5 months postpartum (*P*=.12), fewer children in the WeChat group were given dairy or dairy products.

Proportions of children who were given water were 5.9% (17/287), 3.6% (11/304), and 12.4% (27/217) (0-1 months, 2-3 months, and 4-5 months postpartum, respectively), and no difference was found between the groups. Very few children were given semisolid or solid foods in the first 2 follow-ups; however, the proportion dramatically increased to 22.6% (49/217) 4-5 months postpartum. Similarly, for children being given water, we found no difference between groups at all 3 follow-ups. Therefore, the WeChat intervention in our study could significantly reduce dairy or dairy product supplementation.

### Comparison With Prior Work

It has been demonstrated that information and communication systems, such as web platform, mobile apps, and SMS can be used to promote, educate, and support breastfeeding women, and offer effective means of improving breastfeeding outcomes [33,34]. From 2010 to 2012, a study in Shanghai using SMS to promote breastfeeding found that weekly messages on infant feeding from the third trimester to 12 months postpartum could improve the exclusive breastfeeding rate at 6 months (OR 2.67) but not at 4 months [35]. This explained that many mothers in China were unaware the new concept of exclusive breastfeeding to 6 months of age, and the SMS disseminated the information to mothers [35]. In contrast, the WeChat intervention in our study improved the exclusive breastfeeding rate during the first 2 months postpartum and mainly reduced infant formula instead of water and semisolid or solid food. One reason might be that, in the first 2 months of breastfeeding, mothers may encounter more breastfeeding problems such as perceived insufficient milk supply, breast engorgement, poor suckling technique (of the infant), and sore nipples, which are well-known predictors for early formula supplementation and breastfeeding cessation [36-38]. Therefore, sufficient professional breastfeeding information is needed to increase their breastfeeding confidence during this period. However, 2 months postpartum, mothers may face substantial pressure from family and social culture to introduce formula or to wean [39], which may explain the limited effect on breastfeeding behavior changes afterward.

Improvements in health outcomes rely on putting knowledge into practice; however, population studies have documented that there is a gap between expectations and the actual performance of behaviors in health care and prevention [40]. Compared with the baseline, mothers’ feeding knowledge improved at the 3 follow-ups for both intervention and control groups in this study. Proportions of mothers knowing the duration of exclusive breastfeeding dramatically increased from 28.8% (92/319) at baseline to 69.3% (199/287), 78.9% (240/304), and 89.4% (194/217) (0-1 months, 2-3 months, and 4-5 months postpartum, respectively). In contrast, the exclusive breastfeeding rate dropped from 72.5% (208/287, 0-1 month postpartum) to 44.2% (96/217, 4-5 months postpartum). This is in line with the findings of a previous study [41] that described implementing their breastfeeding promotion interventions in the face of “time and space burdens.” In addition, no differences in mothers’ knowledge were found between the 2 groups, as expected, throughout the study. The reason might be that although mothers in the control group had no access to our WeChat information, they still had various sources of feeding information, including health facilities, mass media, books, and the internet. Successful breastfeeding promotion needs not only education but also support [9,10]. Therefore, multichannel support, such as experts online, through mobilized community, and by health facilities, should also be given to mothers.

With the widespread use of WeChat, it has become a potential health promotion tool in China [22-27,42-44]. The WeChat intervention in our study, providing mothers with information on breastfeeding promotion, could improve breastfeeding practices. The results were in accordance with those of previous studies on weight loss [27] and malaria [23], both of which showed that participants’ knowledge, attitudes, and practices were greatly changed via WeChat official accounts. Information on the benefits of breastfeeding, early initiation, breastfeeding positions, and latching-on were read most during the intervention period, which indicated that these themes were primarily concerned among mothers. However, the effect is unsustainable for a longer time. Therefore, future studies should focus on how to improve the sustainability of the effect.

### Limitations

Our study had several limitations. First, as the Chinese Lunar Year and the COVID-19 outbreak occurred in January 2020 in China, interviewers could not conduct follow-up data collection during that time, which led to a total of 94 mother-child pairs missing the 4-5 months postpartum follow-up deadline (180 days). However, we compared the baseline demographic characteristics between the participants who dropped out and those who were followed up and did not find differences between 2 groups, except for gestational age. Second, given the popular use of WeChat, there are other WeChat official accounts on breastfeeding promotion, and we cannot guarantee that each participant in both groups did not receive breastfeeding knowledge from other sources during the intervention period. However, our randomized controlled trial design could limit such bias. Third, as the randomization unit was individual pregnant women, contamination may exist between intervention and control groups within the same township.

### Conclusions

This health education intervention for promoting breastfeeding via WeChat official accounts was associated with the improvement of exclusive breastfeeding rate in early life. This health education intervention strategy can be used as a reference for local breastfeeding promotion programs, especially, in rural areas of western China where fewer high-quality health services are provided than in urban areas.

## Appendix

Multimedia Appendix 1 Comparison of characteristics between participants with complete and incomplete data.

Multimedia Appendix 2 CONSORT-EHEALTH checklist (V 1.6.1).

## Abbreviations

CONSORT: Consolidated Standards of Reporting Trials
OR: odds ratio
SMS: short message service
UNICEF: United Nations Children’s Fund
WHO: World Health Organization
