# Supplementary material for: Effectiveness of WeChat for Improving Exclusive Breastfeeding in Huzhu County China: Randomized Controlled Trial
Source: J Med Internet Res. 2020 Dec 3;22(12):e23273. doi: 10.2196/23273 (PMC7746496; doi:10.2196/23273)
Supplement: Multimedia Appendix 1 [file jmir_v22i12e23273_app1.docx]

**Supplementary Table Comparison of observed characteristics between participants with complete and incomplete data.**

| **Characteristics** | **First follow-up** | | | **Second follow-up** | | | **Third follow-up** | | |
| --- | --- | --- | --- | --- | --- | --- | --- | --- | --- |
|  | **Followed**  **(n=286)** | **Lost**  **(n=33)** | **P-value** | **Followed**  **(n=304)** | **Lost**  **(n=15)** | **P-value** | **Followed**  **(n=)** | **Lost**  **(n=)** | **P-value** |
| Age (years) (Median (Q1, Q3)) | 28 (25, 31) | 27 (24, 30) | 0.1194 | 28 (25, 31) | 26 (24, 32) | 0.7619 | 28 (25, 31) | 27 (25, 30) | 0.4205 |
| Gestational age (weeks) (Median (Q1, Q3)) | 25 (20, 30) | 25 (18, 32) | 0.9245 | 25 (19, 30) | 28 (20, 30) | 0.4903 | 21 (17, 32) | 28 (26, 30) | <0.0001 |
| 11-27 weeks | 169 (59.1) | 17 (51.5) | 0.4033 | 179 (58.9) | 7 (46.7) | 0.3489 | 144 (66.4) | 42 (41.2) | <0.0001 |
| 28-42 weeks | 117 (40.9) | 16 (48.5) |  | 125 (41.1) | 8 (53.3) |  | 73 (33.6) | 60 (58.8) |  |
| Gravidity |  |  |  |  |  |  |  |  |  |
| [First](https://cn.bing.com/dict/search?q=first&FORM=BDVSP6&mkt=zh-cn) [pregnancy](https://cn.bing.com/dict/search?q=pregnancy&FORM=BDVSP6&mkt=zh-cn) | 62 (21.7) | 10 (30.3) | 0.2618 | 65 (21.4) | 7 (46.7) | 0.0501 | 167 (77.0) | 80 (78.4) | 0.7692 |
| Second pregnancy or above | 224 (78.3) | 23 (69.7) |  | 239 (78.6) | 8 (53.3) |  | 50 (23.0) | 22 (21.6) |  |
| Parity |  |  |  |  |  |  |  |  |  |
| Primipara | 66 (23.1) | 10 (30.3) | 0.3562 | 69 (22.7) | 7 (46.7) | 0.0559 | 167 (77.0) | 76 (74.5) | 0.6321 |
| Multipara | 220 (76.9) | 23 (69.7) |  | 235 (77.3) | 8 (53.3) |  | 50 (23.0) | 26 (25.5) |  |
| Education |  |  |  |  |  |  |  |  |  |
| Primary school or below | 43 (15.0) | 11 (33.3) | 0.0232 | 53 (17.4) | 1 (6.7) | 0.3622 | 40 (18.4) | 14 (13.7) | 0.5744 |
| Middle school | 157 (54.9) | 16 (48.5) |  | 162 (53.3) | 11 (73.3) |  | 116 (53.5) | 57 (55.9) |  |
| High school or above | 86 (30.1) | 6 (18.2) |  | 89 (29.3) | 3 (20.0) |  | 61 (28.1) | 31 (30.4) |  |
| Occupation |  |  |  |  |  |  |  |  |  |
| Housework | 248 (86.7) | 30 (90.9) | 0.8270 | 264 (86.8) | 14 (93.3) | 1.000 | 189  (87.1) | 89 (87.3) | 1.0000 |
| Self-employed | 8 (2.8) | 1 (3.0) |  | 9 (3.0) | 0 |  | 6 (2.8) | 3  (2.9) |  |
| Farmer | 5 (1.8) | 0 |  | 5 (1.6) | 0 |  | 4 (1.8) | 1 (1.0) |  |
| Others | 25 (8.7) | 2 (6.1) |  | 26 (8.6) | 1 (6.7) |  | 18 (8.3) | 9 (8.8) |  |
| Nationality |  |  |  |  |  |  |  |  |  |
| Han | 219 (76.6) | 25 (75.8) | 0.2062 | 231 (76.0) | 13 (86.7) | 0.7209 | 172 (79.3) | 72 (70.6) | 0.2231 |
| Tu | 46 (16.1) | 3 (9.1) |  | 47 (15.5) | 2 (13.3) |  | 30 (13.8) | 19 (18.6) |  |
| Others | 21 (7.3) | 5 (15.1) |  | 26 (8.6) | 0 |  | 15 (6.9) | 11 (10.8) |  |
| Ever received antenatal care | 229 (80.1) | 23 (69.7) | 0.1660 | 240 (78.9) | 12 (80.0) | 1.000 | 174 (80.2) | 78 (76.5) | 0.4476 |
| Anemia | 120 (42.1) | 16 (48.5) | 0.4831 | 132 (43.6) | 4 (26.7) | 0.1966 | 82 (38.0) | 54 (52.9) | 0.0117 |
